# Chronic Sleep Fragmentation Mimicking Sleep Apnea Does Not Worsen Left-Ventricular Function in Healthy and Heart Failure Mice

**DOI:** 10.3389/fneur.2019.01364

**Published:** 2020-01-09

**Authors:** Ignacio Cabrera-Aguilera, Begoña Benito, Marta Tajes, Ramon Farré, David Gozal, Isaac Almendros, Nuria Farré

**Affiliations:** ^1^Unitat de Biofísica i Bioenginyeria, Facultat de Medicina i Ciències de la Salut, Universitat de Barcelona, Barcelona, Spain; ^2^Departament of Human Movement Sciences, Faculty of Health Sciences, School of Kinesiology, Universidad de Talca, Talca, Chile; ^3^Department of Cardiology, Hospital del Mar, Barcelona, Spain; ^4^Heart Diseases Biomedical Research Group, IMIM (Hospital del Mar Medical Research Institute), Barcelona, Spain; ^5^Department of Medicine, Universitat Autònoma de Barcelona, Barcelona, Spain; ^6^CIBER de Enfermedades Respiratorias, Madrid, Spain; ^7^Institut d'Investigacions Biomèdiques August Pi i Sunyer, Barcelona, Spain; ^8^Department of Child Health and Child Health Research Institute, The University of Missouri School of Medicine, Columbia, MO, United States; ^9^Heart Failure Unit, Department of Cardiology, Hospital del Mar, Barcelona, Spain

**Keywords:** sleep apnea, sleep breathing disorders, heart failure, ventricular function, sleep fragmentation

## Abstract

**Aims:** Obstructive sleep apnea (OSA) has been associated with heart failure (HF). Sleep fragmentation (SF), one of the main hallmarks of OSA, induces systemic inflammation, oxidative stress and sympathetic activation, hence potentially participating in OSA-induced cardiovascular consequences. However, whether SF *per se* is deleterious to heart function is unknown. The aim of this study was to non-invasively evaluate the effect of SF mimicking OSA on heart function in healthy mice and in mice with HF.

**Methods and Results:** Forty C57BL/6J male mice were randomized into 4 groups: control sleep (C), sleep fragmentation (SF), isoproterenol-induced heart failure (HF), and mice subjected to both SF+HF. Echocardiography was performed at baseline and after 30 days to evaluate left ventricular end-diastolic (LVEDD) and end-systolic (LVESD) diameters, left ventricular ejection fraction (LVEF) and fraction shortening (FS). The effects of SF and HF on these parameters were assessed by two-way ANOVA. Mice with isoproterenol-induced HF had significant increases in LVEDD and LVESD, as well as a decreases in LVEF and FS (*p* = 0.013, *p* = 0.006, *p* = 0.027, and *p* = 0.047, respectively). However, no significant effects emerged with SF (*p* = 0.480, *p* = 0.542, *p* = 0.188, and *p* = 0.289, respectively).

**Conclusion:** Chronic SF mimicking OSA did not induce echocardiographic changes in cardiac structure and function in both healthy and HF mice. Thus, the deleterious cardiac consequences of OSA are likely induced by other perturbations associated with this prevalent condition, or result from interactions with underlying comorbidities in OSA patients.

## Introduction

Sleep apnea is the most frequent sleep breathing disorder in patients with heart failure (HF), and is strongly associated with worse prognosis (1). The two major physiological perturbations experienced by patients with sleep apnea are intermittent hypoxia and sleep fragmentation (SF), and are a consequence of the recurrent events of nocturnal airway collapse. These two characteristic perturbations have been mechanistically implicated in the mid-term and long-term adverse consequences of OSA. However, whereas the effects of recurrent hypoxemia in the healthy heart or in HF have been extensively studied (2, 3), there is no specific information on the potential cardiac effects induced by chronic SF. Given that increased sympathetic activation is common in both HF and SF (4, 5), it is possible that SF negatively contributes to cardiac function in the healthy heart, and more particularly in HF.

The aim of this work was to assess whether chronic SF affects ventricular cardiac function in a mouse model mimicking sleep apnea. To this end, we used non-invasive cardiac echography since this technique is immediately applicable to routine patient assessment.

## Methods

The experimental protocol (approved by the institutional Ethical Board) was conducted on 40 male mice (C57BL/6J; 10 weeks old; 12 h light/dark cycle; water/food *ad libitum*) randomly distributed into 4 groups (*n* = 10 each). In two groups the mice were allowed to sleep normally: healthy control (C) and heart failure (HF). In two similar groups (C+SF, HF+SF), SF mimicking sleep apnea was imposed.

HF was conventionally induced by continuous infusion of isoproterenol (6). Briefly, mice in HF and HF+SF groups were anesthetized by isoflurane inhalation and an osmotic mini-pump (Alzet, model 1004) was implanted subcutaneously in the flank. The pump was set to deliver 30 mg/kg per day of isoproterenol (Sigma Aldrich; in sterile 0.9% NaCl solution) for 28 days. Buprenorphine (0.3 mg/kg, i.p.) was administered 10 min before surgery and after 24 h, and the suture was removed 7 days after surgery. Healthy animals (C, C+SF) were subjected to the same protocol with the only difference that no isoprotenerol was dissolved into the 0.9% NaCl pump medium.

Two days after surgery, SF was daily applied by means of a previously described and validated device for mice (Lafayette Instruments, Lafayette, IN) based on intermittent tactile stimulation (7, 8). Sleep arousals were induced by a mechanical near silent motor with a horizontal bar sweeping just above the cage floor from one side to the other side in the standard mouse laboratory cage. This automatic system, with no human intervention, minimized stress to the animal. To apply SF mimicking sleep apnea, 2 min intervals between each sweep (i.e., corresponding to an apnea-hypopnea index of 30 events/h) were applied during the murine sleep period (8 a.m. to 8 p.m.) for 28 days (until day 30 from surgery).

Echocardiography (Vivid IQ and L8-18i-D Linear Array 5–15 MHz, General Electric Healthcare, Horten, Norway) was performed in each animal at baseline, before surgery, and at day 30 following a standard protocol (9). The following echographic indices were subsequently computed: left ventricular end-diastolic (LVEDD) and end-systolic diameter (LVESD), left ventricular ejection fraction (LVEF) and fraction shortening (FS). All echocardiographic measurements and computations were carried out by a single operator (NF), who was blind to the experimental group.

Immediately after the final echography, blood was extracted to measure circulating interleukin (IL)-6 (ELISA kit Ab100712, Abcam), which can be considered a systemic biomarker of SF effects (10), and animals were euthanized by exsanguination.

All data are presented as mean ± SEM. Comparison of echocardiographic data between all groups at baseline was performed using one-way ANOVA. Comparison of IL-6 plasma concentrations and echocardiographic data between all groups at day 30 was performed using two-way ANOVA followed by the Student-Newman-Keuls comparison method. For all tests, *p* < 0.05 was considered statically significant.

## Results

**Figure 1:**
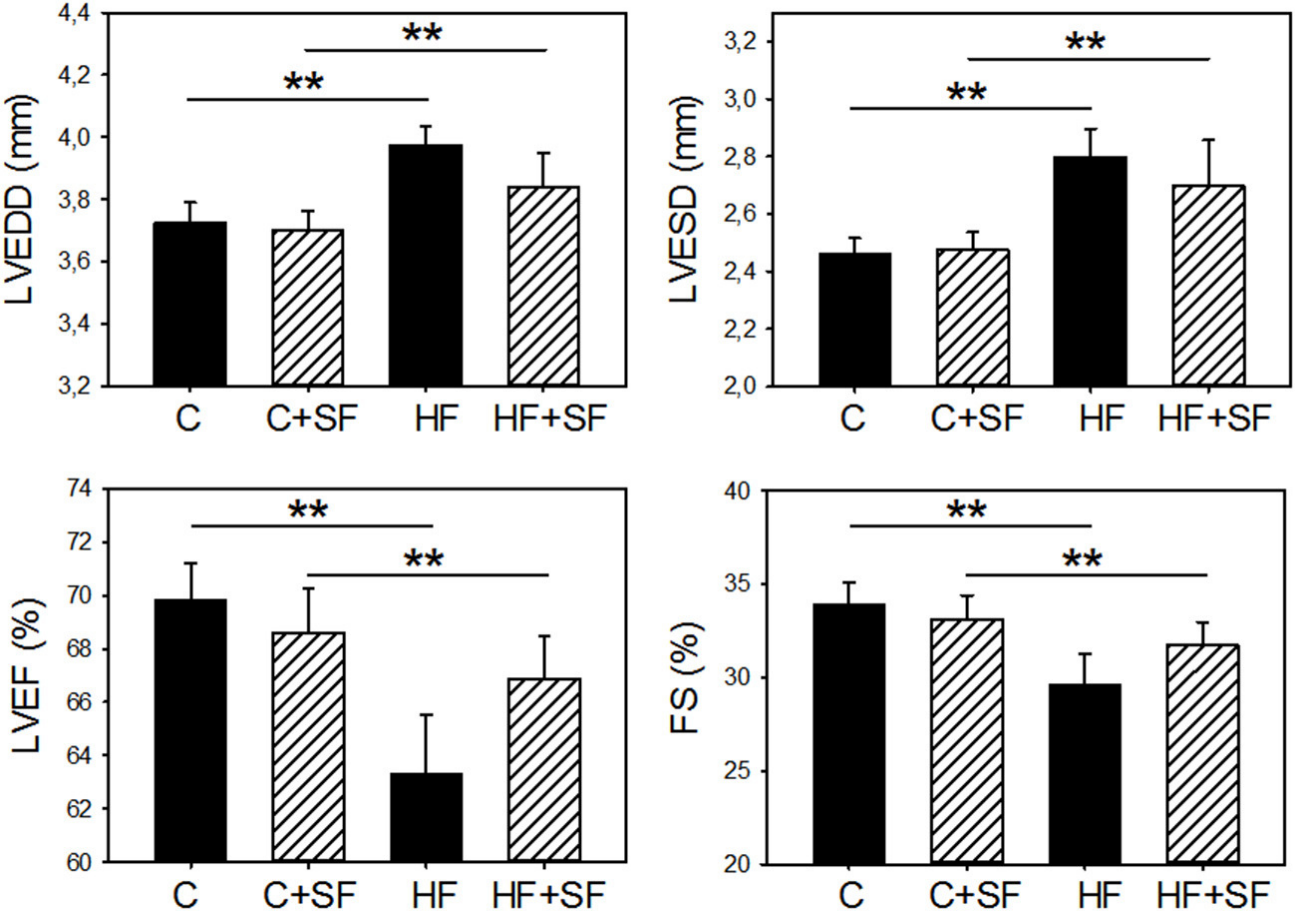
Sleep fragmentation (SF) mimicking sleep apnea did not modify left ventricular echocardiographically-measured structure and function in control healthy mice (C) and in heart failure (HF) mice. LVEDD, end-diastolic diameter; LVESD, end-systolic diameter; LVEF, left ventricular ejection fraction; FS, fraction shortening. Only HF significantly changed cardiac indices in both normal sleep and in SF (**p < 0.05), with no significant differences induced by SF in healthy controls (C) and in HF mice.

First, as expected from random distribution of mice into the four experimental groups, there were no significant differences in the baseline values of LVEDD, LVESD, LVEF, and FS (*p* = 0.308, 0.756, 0.817, and 0.771, respectively). Second, effective application of SF was confirmed by a 10-fold (*p* < 0.001; 2-way ANOVA) increase in circulating IL-6 in the mice subjected to this challenge [82.2 ± 27.0 pg/ml (C-SF) and 62.2 ± 36.3 pg/ml (HF+SF)], as compared with control sleep mice [9.1 ± 6.4 pg/ml (C) and 6.3 ± 2.8 pg/ml (HF)]. Third, isoprotenerol infusion effectively induced HF. As expected from extensive data in the literature (6), mice in the HF groups had significant increases in LVEDD and LVESD as well as significant reductions in LVEF and FS (Figure 1). Indeed, 2-way ANOVA of the 4 groups showed that HF was significant (*p* = 0.013, 0.006, *p* = 0.027, and *p* = 0.047 for LVEDD, LVESD, LVEF, and FS, respectively).

The main finding however, was that SF did not significantly worsen ventricular function (LVEDD, LVESD, LVEF, and FS), neither in healthy mice, nor in animals with HF (Figure 1), as indicated by 2-way ANOVA (*p* = 0.294, *p* = 0.657, 0.509, and *p* = 0.625, respectively). Moreover, there were no statistically significant interactions between factors HF and SF (*p* = 0.480, *p* = 0.542, 0.188, and *p* = 0.289, respectively).

## Discussion

Although sleep apnea considerably increases the morbidity and mortality of cardiovascular diseases (1), the potential mechanisms are poorly understood. Whereas, the deleterious role of intermittent hypoxemia has been widely investigated, the cardiac effects of SF, the other main physiological perturbation in sleep apnea, has not been specifically explored to date. For the first time, this study provides experimental data showing that SF mimicking sleep apnea does not worsen cardiac function. Interestingly, this lack of effect of SF was seen both in healthy mice and in animals with HF.

We conducted the study in an isoproterenol-induced heart failure model since the involved mechanism [increased sympathetic activation (11)] could be synergistic with the physiological response to SF. Indeed, sleep fragmentation is considered a main determinant of the sympathetic activation in sleep apnea, independently of the frequency and severity of oxygen desaturation (4). It should be mentioned, however, that animals in the HF model could have some sleep alterations since it has been reported that direct infusion of isoproterenol into the lateral hypothalamic area may result in increased arousability (12). However, it is not possible to compare the doses directly injected into the lateral hypothalamic area with the ones that correspond into this area from the concentration of the intraperitoneally injected isoproterenol in the model of SF. Notwithstanding, to the best of our knowledge no sleep alterations have been reported from the large number of studies using the isoproterenol-induced heart failure model. Hence, this question deserves further investigation, for instance by EEG-based sleep characterization of animals subjected to this drug-induced heart failure model. However, contrary to what would be expected, our study showed that SF mimicking sleep apnea did not affect cardiac function.

It is interesting to compare our results on SF with previous data on intermittent hypoxia. Studies carried out in C57BL/6J and HF-transgenic mice reported that 28 days of intermittent hypoxia resulted in altered heart function, as measured by echocardiography. Surprisingly, intermittent hypoxia was not deleterious, and in fact resulted in improvements in cardiac function (2, 3). In this setting, we did not find any changes induced by SF, even though application of SF of similar duration has been shown to be deleterious to several organs other than the heart (7, 8, 10, 13). However, we cannot rule out that a longer exposure to SF could be required to manifest cardiac function alterations. The fact that physiological perturbations characterizing sleep apnea (SF and recurrent hypoxemia) are not deleterious to cardiac function is intriguing. Besides the need for expanded research on this issue, the cumulative findings to date may account for some of the contrasting results reported by clinical studies addressing the interaction between sleep apnea and heart diseases. Indeed, in obesity hypoventilation syndrome, the prevalence of chronic heart failure had the strongest negative association with the highest tertile of oxygen desaturation index (14). More intriguingly, treatment of severe central sleep apnea with adaptive servo-ventilation in patients with HF was associated with an increase in all-cause and cardiovascular mortality (15).

Our experimental study has some limitations. Our measurements do not completely preclude an adverse effect of SF on the heart, since it is still possible that diastolic function could be affected. However, the few studies that have reported changes in diastolic function and sleep abnormalities involved acute sleep deprivation (16), and not SF. The possibility also remains that SF could worsen HF of different etiologies, even if no such effects were apparent in our HF model. Accordingly, studying how SF interacts with HF in models induced by different challenges (e.g., pressure/volume overload or ischemic injury) (17) is warranted. In addition, we evaluated ventricular function by echocardiography to assess for clinically relevant structural and functional changes. However, we cannot rule out whether SF induces minor changes at the molecular and cellular level in the heart. It is also worth noting that our study was carried out in male animals exclusively. Taking into account that some sex differences have been reported in rodent heart failure models (18), future studies addressing the interaction between SF and HF should be also carried out in female mice.

In conclusion, SF did not induce echocardiographic detectable deleterious effects in heart structure and function in a sleep apnea model imposed on both healthy and HF mice. These findings suggesting that the potential cardiac consequences of sleep apnea in patients are mainly induced by other perturbations resulting from the obstructive nocturnal events or reflect interactions with the underlying comorbidities frequently found in patients with sleep apnea.

## Data Availability Statement

All datasets generated for this study are included in the article/supplementary material.

## Ethics Statement

The animal study was reviewed and approved by Ethical Committee for Animal Research of the University of Barcelona.

## Author Contributions

IC-A carried out the experiments, analyzed the data, and contributed to draft the manuscript. BB, MT, RF, and DG contributed to analyze the data and revise the manuscript. NF and IA conceived the work. NF carried out measurements, drafted the manuscript, and supervised the study.

### Conflict of Interest

The authors declare that the research was conducted in the absence of any commercial or financial relationships that could be construed as a potential conflict of interest.
